# A *ccpp-6* deletion mutation does not impair gross cilia integrity in *C. elegans*

**DOI:** 10.17912/micropub.biology.000649

**Published:** 2022-10-09

**Authors:** Jessica Dominguez, Bhumi P Shah, Nina Peel

**Affiliations:** 1 The College of New Jersey, 2000 Pennington Rd, Ewing, NJ 08618

## Abstract

Tubulin glutamylation is a reversible modification that regulates microtubule function in cilia. The removal of glutamylation from microtubules is carried out by a family of cytosolic carboxypeptidase (CCP) enzymes. *C. elegans* has two deglutamylating enzymes, CCPP-1 and CCPP-6, homologs of mammalian CCP1 and CCP5 respectively. CCPP-1 is required for ciliary stability and function. To determine whether CCPP-6 is similarly required for cilia integrity in *C. elegans* we analyzed the *ccpp-6(ok382)* deletion mutant. We find that both dye-filling and male mating are normal, suggesting that CCPP-6 is not required for ciliary integrity in *C. elegans.*

**Figure 1. Markers of cilia function are unaltered in ccpp-6(ok382) mutants.**
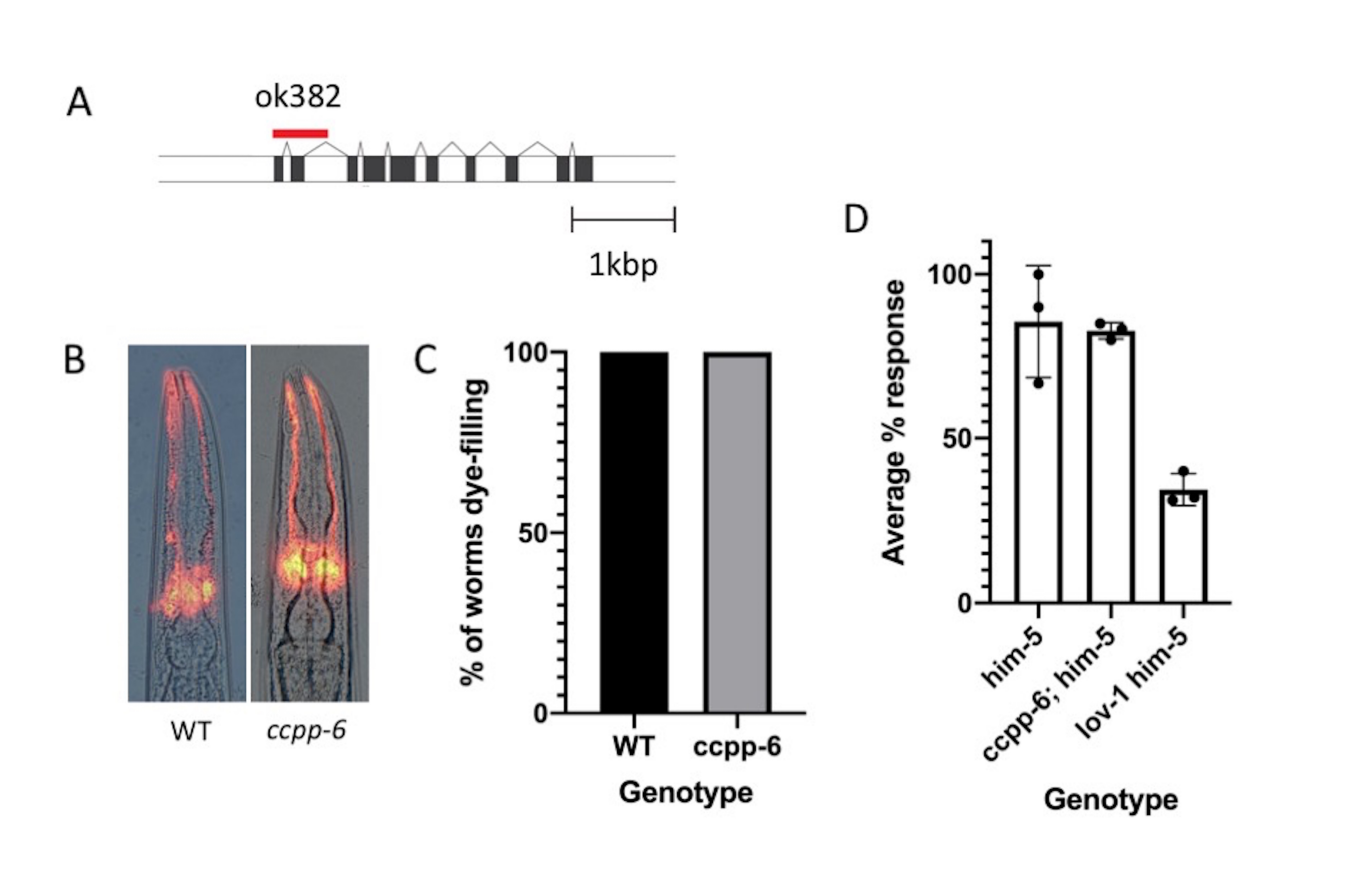
A) Diagram of the ccpp-6 gene structure indicating the location of the *ok382* deletion (red), coding exons in black; Scale bar: 1kbp. B) images of dye filling, and C) quantification of numbers of worms that filled with dye. Number of worms analyzed: WT n=43; *ccpp-6* n=70. D) Percentage of males completing the response step of male mating. Response rate for three independent trials shown (·), and the mean of the replicates indicated. Error bars indicate s.d.

## Description

The microtubules of the ciliary axoneme are subject to a variety of post-translational modifications, which regulate cilia function and structural integrity. One such modification, glutamylation, involves the reversible addition of glutamic acid to the C terminal tail of tubulin. Both hypo- and hyper-glutamylation have been associated with ciliary dysfunction in *C. elegans* (O’Hagan *et al.* 2011; Chawla *et al.* 2016). Loss of the deglutamylating enzyme, CCPP-1, causes a dye-filling phenotype indicative of a progressive degeneration of amphid and phasmid cilia. In male-specific neurons PKD-2 is mislocalized when CCPP-1 is lost, leading to male mating defects (O’Hagan *et al.* 2011, 2017). In addition to CCPP-1, *C. elegans* possesses a second cytosolic carboxypeptidase, CCPP-6. Similar to CCPP-1, CCPP-6 is expressed in the amphid neurons *,* moreover the *ccpp-6(ok382)* mutation leads to hyperglutamylation, indicating that CCPP-6 has deglutamylating activity (Kimura *et al.* 2010). We analyzed amphid and male-specific cilia in the *ccpp-6(ok382)* mutant worms to determine whether CCPP-6, like CCPP-1, is required for cilia integrity.

We first obtained and outcrossed the *ccpp-6(ok382)* deletion mutant. The *ok382* deletion removes the start codon of the gene and both the first and second exons in their entirety (Fig 1A). The *ccpp-6(ok382)* worms did not show a defect in dye-filling with dye detected in 100% of adult amphid cilia (Fig1B&C), suggesting that the cilia are structurally intact. This contrasts with *ccpp-1* mutants, where adult worms do not uptake dye due to structural defects in the cilia (O’Hagan *et al.* 2011). Loss of CCPP-1 also leads to defects in the male-specific CEM cilia and an inability to complete the response step of male mating (O’Hagan *et al.* 2011, 2017). We therefore analyzed male mating in the *ccpp-6(ok382)* mutants (Fig 1D). The *lov-1; him-5* strain is known to have a defect in male mating and was used as a control. We found no significant difference between the *him-5* control strain, which does not have a mating defect, and the *ccpp-6; him-5* strains (student’s *t* -test P>0.05) indicating that CCPP-6 is not required for proper male mating response and thus the cilia are likely structurally intact.

In summary, we have found that the *ccpp-6(ok382)* mutants do not have overt defects in the amphid cilia nor impairment of the cilia required for male mating. Because the *ok382* deletion allele removes the start codon (fig 1A) and leads to hyperglutamylation of the ciliary microtubules (Kimura *et al.* 2010) we infer that this allele severely diminishes CCPP-6 function. Thus the lack of ciliary impairment in our assays suggests that the CCPP-6 protein is dispensable for gross ciliary function, although we cannot rule out the presence of subtle defects. This contrasts with CCPP-1 which is absolutely required for the integrity of both amphid and male-specific cilia (O’Hagan *et al.* 2011, 2017).

## Methods

We carried out the response assay as detailed in (Chawla *et al.* 2016). Briefly, young males were isolated and kept at 15°C overnight, and warmed to room temperature before use. Plates were seeded with 10μl of concentrated OP50, and ~30 *unc-119* hermaphrodites placed in the food. Two males were placed in the center of the plate, and individually monitored for the execution of the response step of male mating during a 5 min period. A male was scored as positive if it began scanning a hermaphrodite with his tail and maintained contact for 10s or more. The response rate for each of three independent trials is shown, and the average of the three trials is plotted. Each trial assayed at least 10 worms of each genotype.

## Reagents

**Table:** 

**Strain name**	**Genotype**	**Available from**
NIN59	*ccpp-6(ok382) II*	authors
NIN81	*ccpp-6(ok382) II; him-5(e1490) V*	*authors*
PS3151	*lov-1(sy552) II; him-5(e1490) V*	*CGC*
DR466	*him-5(e1490) V*	*CGC*
HT1593	*unc-119(ed3 )III*	*CGC*
